# The impact of a coach-guided personalized depression risk communication program on the risk of major depressive episode: study protocol for a randomized controlled trial

**DOI:** 10.1186/s12888-024-06393-9

**Published:** 2024-12-18

**Authors:** JianLi Wang, Cindy Feng, Mohammad Hajizadeh, Alain Lesage

**Affiliations:** 1 Department of Community Health & Epidemiology, Faculty of Medicine, Dalhousie University, Halifax, Canada; 2 Department of Psychiatry, Faculty of Medicine, Dalhousie University, Halifax, Canada; 3 School of Health Administration, Dalhousie University, Halifax, Canada; 4 Department of Psychiatry, Faculty of Medicine, University of Montreal, Montreal, Canada; 5 5790 University Ave, Halifax, NS B3H 1V7 Canada

**Keywords:** Major depression, Randomized controlled trial, High risk, Self-help, Coach-guided, Risk communication

## Abstract

**Background:**

Depression is a highly prevalent and disabling mental health problem. Self-help has been strongly advocated for dealing with depression. Built upon the research on risk prediction modeling and risk communication, we developed a coach-guided, personalized depression risk communication tool (PDRC) for sharing information about individualized depression risk and evidence-based self-help strategies. The primary objective of this project is to evaluate the impact of the PDRC on the 12-month risk of major depressive episode (MDE) in Canadians who are at high risk of MDE.

**Methods:**

This is an assessor-blinded randomized controlled trial (RCT) with two arms. We will recruit 500 males and 500 females in the communities across the country. Individuals are eligible, if they: (1) are 18 years or older, (2) have not had a depressive episode in the past two months, (3) are at high risk of MDE based on the sex-specific risk predictive algorithms for MDE (predicted risk of 6.5% + for men and of 11.2% + for women), (4) can communicate in either English or French, and (5) agree to be contacted for follow-up interviews. After screening and baseline assessment, participants will be randomized by sex into intervention and control group in a 1:1 ratio. Participants in the intervention group will receive the coach-guided PDRC. The participants are assessed at baseline, 3 and 12 months via computer assisted telephone interview system, regarding the presence of MDE, depressive and anxiety symptoms, use of self-help strategies, mental health services use and self-efficacy.

**Discussion:**

The coach-guided PDRC may empower users to actively engage in self-management, leading to reduced risk of MDE. If successful, the coach-guided PDRC will lead to a novel selective prevention program that is closely aligned with the tiered mental health services model, contributing to early prevention of depression and better mental health wellbeing.

**Date of trial registration:**

: 2024-10-02.

**Protocol version and date:**

December 6, 2024. Version #1.

**Trial registration:**

: NCT06619366.

**Supplementary Information:**

The online version contains supplementary material available at 10.1186/s12888-024-06393-9.

## Background

Depression is a highly prevalent and disabling mental health problem [1]. In 2012, the annual prevalence of major depressive episode (MDE) was 4.8% [2] in the Canadian general population; the prevalence was higher in females (5.0%) than in males (2.9%) [2]. In the past two decades, there has been no measurable change in the prevalence of MDE in Canadians [3], which was consistent with the findings in other countries [4]. The unchanged prevalence of MDE calls for early preventive interventions to reduce the incidence of MDE [5], e.g., identifying individuals who are at high risk (i.e., selective prevention) or those with subsyndromal symptoms (i.e., indicated prevention) [6] and intervene to stop MDE before it happens.

Since 2008, drawing substantively upon the experience in the United States [7] and the United Kingdom [8], some Canadian provinces have adopted a tiered model to design and deliver mental health and addiction services [9–11]. In this tiered model (Supplement Fig. 1), Tier 1 includes population-based health promotion, education and prevention interventions targeted at the general population and/or at-risk populations with the objective of promoting healthy lifestyles and prevention. Tier 2 focuses on early intervention & self-management interventions targeted to people at risk, including self-help. The top three tiers are composed of mental health and addiction (MHA) specialty services. Systematic reviews [12, 13] of indicated prevention programs for depression have reported encouraging results, with some programs appeared to alleviate depressive symptoms and/or boost productivity. These indicated prevention programs are often in standardized formats with multiple sessions learning cognitive behavioral therapy (CBT) or problem solving therapy skills [13, 14]. However, many of these programs either require in-person attendance at a purpose-specific intervention meeting, which can be a barrier for those with a busy schedule, or web-based programs that often have sub-optimal adherence and fidelity [15]. Furthermore, these indicated prevention programs were designed for and evaluated in individuals with sub-clinical depression, not those who are at high risk of having depression (e.g., exposure to a set of risk factors, such as personal and family history of depression, low socioeconomic status, negative life events, childhood trauma) but at not qualified for specialty mental health services. Personalized intervention programs targeting these high-risk populations in the community (i.e., those fit in Tier 1 and/or 2), that can be quickly delivered by phone or video conference at the time in need, remain lacking [16]. We developed a coach-guided personalized depression risk communication (PDRC) tool to fill this knowledge gap.

Risk communication is the open two way process of informing individuals about the factors that can increase their risk for a health condition and the steps they can take to reduce that risk [17]. Risk communication goes beyond simply sharing information about individualized risk; it also shares possible actions to be taken [18]. The notable clinical guidelines for coronary heart disease [19, 20] and the Institute of Medicine report [21] recommend that individuals should know their personal risk, and high-quality risk communication tools should be developed. Currently, personalized risk communication is happening on a large scale for preventing cardiovascular diseases, diabetes and cancer [22]. There is a paucity of research on risk communication for mental health problems. Risk communication is particularly important for people with an existing mental health problem, or at high risk of experiencing a mental health problem, because people who are more aware of their emotions can prepare themselves more effectively for the process of changing their thoughts, beliefs, and schemas [23]. Notably, the aim of the PDRC we developed is not only to share personalized risk information, but also motivate people to engage in evidence-based self-help strategies that are suitable for them.

Risk communication in oncology and cardiology are delivered either through written materials or web, or with professional guidance (e.g., in-person or telephone education and counselling by trained coaches or health professionals) [24, 25]. In the field of mental health, the evidence of risk communication is scarce. The only research related to risk communication was a randomized controlled trial (RCT) in the Spanish primary care setting, in which depression risk was communicated to patients by family doctors via face-to-face conversations [26]. We know from a meta-analysis that guided self-help is more effective than un-guided self-help for managing depression [27] Particularly, the effectiveness and comprehensiveness can be further enhanced if the guided risk communication is delivered based on the principles of motivational interviewing (MI). MI aims to enhance an individual’s motivation and commitment to a specific goal by uncovering and examining their own reasons for change, all within a supportive and compassionate environment [28].

In our RCT, we will employ the principles of MI to enhance the effectiveness of risk communication. Unlike the RCT in the Spanish primary care setting, the target population of the proposed study are the general population in the community, not from any specific medical settings. The primary research question to be answered by this RCT is: **Can the coach-guided PDRC reduce the risk of MDE**? The secondary research questions include:

1. What is the impact of receiving the coach-guided PDRC on self-help and help seeking behaviors?
2. What is the impact of receiving the coach-guided PDRC on depressive symptoms?
3. Does the effect of coach-guided PDRC differ by levels of self-efficacy? and.
4. What is the cost-effectiveness of the coach-guided PDRC?

## Methods/Design

This is an assessor-blinded RCT with two arms. Participants will be recruited from communities across the country. After screening and baseline assessment, participants will be randomized into intervention and control groups in a 1:1 ratio. Participants in the intervention group will receive the coach-guided PDRC. This RCT has received approval from the Health Research Ethics Review Committee of Dalhousie University (REB # 2024–7208). The trial is registered at the ClinicalTrials.gov.

### Inclusion criteria

(1) no MDE at baseline, or full remission for 2 months for those who had a past MDE (see below the question). (2) Aged 18 and 65 years. (3) At high risk of MDE based on the depression risk calculators (predicted risk of 6.5%+ for males and 11.2%+ for females) [29], which represent the top two deciles of male and female populations in Canada [29] (4) Agreement to be contacted for follow-up assessments, and (5) no language barriers to English or French. The status of remission will be assessed by the question: “In the past 2 months or longer, has your mood been much improved or back to normal AND you DIDN’T have the symptoms?” This question was adopted from the US NESARC study [30]. Although the risk calculators are sex-specific, our recruitment will be sex and gender inclusive; the recruitment is open to all sexes and genders.

### Exclusion criteria

Individuals who (1) cannot provide informed consent, (2) do not agree to be followed, (3) cannot communicate in English and French, and (4) report suicide behaviors (score > 0 on item 9 of the Patient Health Questionnaire (31) PHQ-9). Individuals with suicide behaviours should be directed for medical attention.

*The intervention* has two integrated components: PDRC and coach-guidance. The PDRC has five sections: (1) individualized depression risk, the interpretation of the risk information, and the national average risk. (2) Factors contributing to the risk. These are the modifiable risk factors the person is currently exposed to, based on the MVRPs and participant self-report. (3) A graphical presentation of their risk and national average risk, and (4) Evidence-based self-help strategies the person currently engages in, and the self-help strategies [32] the person is encouraged to use. The PDRC will be sent to participants by email/mail within two days after the baseline and follow-up assessments.

The coach will contact the participant within a week after the PDRC is provided by telephone or video conference, depending on participants’ preference. The role of the coach is to assist the participant in understanding and interpreting the PDRC, answering their questions, and helping identify self-help strategies that are feasible for the participant; they do not provide psychotherapy. The coaching session will follow the four processes of MI [28]: (1) engaging participants in a mutually trusting and respectful working relationship, (2) focusing on the goals for risk communication, (3) evoking and strengthening motivation for taking self-help strategies, and (4) planning and implementing self-help strategies. A coaching manual has been developed to ensure coaching fidelity, and the fidelity level in our pilot RCT was 97%. The coaching sessions will be audio recorded and reviewed at weekly team meeting to ensure coaching fidelity, with 20% of the sessions be rated by two independent raters for reliability. The coaching manual covers the elements that are essential to include (e.g., asking for commitment) and not include (e.g., providing unsolicited advice) during the coaching sessions. The same procedures will be used for follow-up coaching sessions at 3 months. Communications beyond the scheduled coaching will not be allowed to ensure that participants receive same type of intervention.

### The control group

Participants in the control group will receive their individualized depression risk information. Receiving individualized depression risk information is considered as “control” because: (1) our previous RCT showed that receiving individualized depression risk information caused no psychological harm and was effective in reducing distress and improving function [33]; (2) receiving depression risk information was used as control in our pilot study; (3) the pilot study suggested that the coach-guided PDRC may be more effective than receiving depression risk information alone.

### Procedures of recruitment, screening and randomization

The *recruitment*,* screening*,* baseline assessment* and *randomization* will be contracted to a telephone interview firm that has access to household telephone numbers across the country, validated cellphone number database, and databases for specific demographics, and that can conduct interviews in English and French. The firm will first allocate a set of phone numbers (e.g., 100,000), and call the numbers randomly, until the target sample size is reached. The interviewers will explain the study objectives and procedures and answer questions. Potentially eligible participants will be sent a web survey link that can be accessed via a computer or mobile phone. Participants need to provide consent before completing the survey. The consent will be registered online and transferred to the research team. Follow-up interviews at 3 and 12 months will be conducted via the computer assisted telephone interview system (CATI) by interviewers at the principal investigator’s laboratory at Dalhousie University. Oral consent will be obtained and audio recorded before each follow-up interview. The recruitment is scheduled to start on January 15th 2025 and complete around August 31st, 2025.

We acknowledge the limitations associated with different recruitment methods. Given that the target population of this study are adults who are at high risk of having a MDE in Canada and a sampling frame of the target population does not exist, the proposed recruitment, screening and data collection methods are a feasible and economical way for the proposed study, and we used the same approach for our previous RCTs [34, 35] and the pilot study. We will recruit participants proportional to the sex and age distributions of the high-risk population as defined in the section of “Inclusion criteria” to minimize the possibility of selection bias.

#### Screening

Once a potentially eligible participant is identified, the interviewer will confirm the participant’s age and administer the lifetime version of the Composite International Diagnostic Interview – Major Depression (CIDI) [36] MDE module and the sex-specific MVRPs to assess eligibility. Interviewees who have a MDE in the past 2 months or are below the risk thresholds based on the MVRPs, will be excluded. Individuals with an ongoing MDE will be encouraged to contact their family doctors and information about local mental health resources will be provided. For those who are at low risk, the website of the MVRPs will be provided so they may monitor their risk in the future.

*Baseline assessment* will include demographic and socioeconomic status, depressive and anxiety symptoms, physical illness, self-help behaviours, mental health services use and self-efficacy.

*Randomization* will be carried out immediately after baseline assessment, in male and in female participants separately because the MVRPs are sex-specific and the thresholds of high-risk differ by sex. Most survey firms use a survey software containing a random number generator which randomly creates a digit. This randomization approached was used in our previous RCT, and the intervention and control groups of the trial were balanced in baseline characteristics [33].

### Blinding

The survey firm will securely transfer encrypted baseline data to the project coordinator on a daily basis, with the group assignment data in a separate file. The follow-up assessments will be conducted at the principal investigator’s (PI) telephone interview laboratory at Dalhousie University. Over the study period, investigators will be blinded to participants’ group status. The survey firm interviewers who conduct baseline assessment and randomization, will not be involved in follow-up interviews. The interviewers who conduct the follow-up interviews will not have access to participants’ group status. The coaches will not be involved in follow-up assessments. Given our description of study objectives, participants may know their group status. After the 12-month interview, group status will be linked with interview data by study ID numbers. At the follow-up assessments, if participants develop an MDE, they will be encouraged to contact their family doctor and information about local mental health resources will be provided.

### Follow-up assessments

Participants will be followed and assessed at 3- and 12-month so we can assess the short- and mid-term effects of the intervention. The follow-up assessments will be conducted via telephone. After each assessment, the following materials will be mailed to all participants of the two groups: (1) a thank-you letter, including the date of the next interview, and (2) $20 incentive as appreciation for their participation. Participants in the control group will receive the same package as those in the intervention group.

### Strategies for reducing attrition

1. Well trained interviewers to build and maintain rapport with participants.
2. Be responsive to participants’ questions.
3. Sending interim reports to participants.
4. Providing financial incentives for participation.

### Data monitoring

The PI and the research coordinator (RC) are responsible for daily operation of the project, and monitoring data collection, data quality and potential adverse events. The PI and RC report to the research team at teleconferences held every 3 months. The funder plays no role in the process of data monitoring.

### Adverse events/harm

Our study population are individuals who are not in an episode of depression but are at high risk. The data collection is conducted via telephone. Therefore, the possibility of physical injuries is minimum. In the circumstance that the participant may need mental health services, the interviewers are instructed to encourage the individual to seek professional help and provide information about local mental health resources. If an unintended adverse event occurs, the ERB will be immediately notified and the event will be jointly reviewed by the board and the research team.

### Primary and secondary outcome measures

*The primary outcome* is the *proportion of MDE at 12-months*, measured by the 12-month version of CIDI-MD [36]. This version of CIDI is a fully structured diagnostic interview for mental disorders in the past 12 months based on the DSM-IV criteria, and has been widely used in population mental health surveys conducted by Statistics Canada and by others [36].

#### *The secondary outcomes* include

(1) *Depressive symptom score at 3 and 12 months*, measured by the PHQ-9 [31]. The PHQ-9 scores each of the 9 depressive symptoms in the past two weeks as “0” (not at all) to “3” (nearly every day) with total scores ranging from 0 to 27. The PHQ-9 has excellent internal reliability (Cronbach’s α = 0.89) [31]. (2) C*hange in self-help score* measured by the Self-management Strategy Use Scale (SSUS). The SSUS assesses the frequency of using each of the 14 evidence-based self-help strategies. Frequency of use is rated on a 5-category scale. The SSUS has good internal consistency (Cronbach’s α = 0.80). (3) *Mental health services use* over the study period. Standard questions used by Statistics Canada about in-patients and out-patient psychiatric care, ED mental health services use, and contacting physician and other health professionals for mental health and emotional problems. (4) *Sick leave and productivity* measured by the WHO’s Health and Work Performance Questionnaire (HPQ) which collects information about number of sick leave days and presenteeism in the past 30 days [39]. The HPQ had good concordance with the corporate archival productivity measures [39].

*Other measures* include age, biological sex at birth (male, female, intersex, other), gender [man, woman, Indigenous or other cultural identities (e.g., two spirit), others (e.g., gender fluid, non-binary)] [37] race/ethnicity, marital status, employment status, and levels of education and income, number of hours for caring family members each week, history of MDE, symptoms of anxiety, comorbid physical health conditions and self-efficacy. *Anxiety symptoms* will be measured by GAD-7 [38]. *The GAD-7* is a valid and efficient tool for screening for generalized anxiety disorder and assessing its severity in clinical practice and research. A higher score indicates more severe anxiety symptoms. The internal consistency of the GAD-7 was excellent (Cronbach α = 0.92). Test-retest reliability was also good (intraclass correlation = 0.83) [38]. *Self-efficacy* will be measured using the General Self-Efficacy scale [40, 41]. The lifetime version of the CIDI will be administered at baseline to ascertain *history of MDE.*

The collected data will be stored at the secured cloud drive of Dalhousie University. Only the interviewers and coaches will have access to individual data. Only de-identified data will be used for data analysis and only aggregated data will be published and presented.

### Statistical analysis

As the intervention is conducted in males and females separately, data will be initially analyzed in males and females separately. We will first compare the groups in baseline demographics, socioeconomic and clinical characteristics, and missing values. To follow the CONSORT guidelines for reporting RCTs [42] we will perform both complete case and intent-to-treat (ITT) analyses to allow readers to interpret the effect of the intervention. In ITT analysis, we will include all participants with the group status they are originally assigned. Following the recommendations published in the N Engl J Med [43], and BMJ [44], missing values will be imputed using multiple imputation with 20 imputed datasets.

The proportions of MDE at 12 months (primary objective) and at 3 months will be estimated and compared between the intervention and control groups. For the primary objective, mixed-effects logistic regression modeling will be performed using combined imputed datasets based on Rubin’s rules, adjusting for the effects of demographic and socioeconomic characteristics, baseline PHQ-9 score, predicted risk of MDE, caregiving burden, and comorbid physical conditions [45]. Additionally, it will account for within-participant correlation over time through the incorporation of random effects. Within-and between‐group effect sizes will be estimated by computing Cohen’s h [46]. The analyses will be repeated among complete cases. Sensitivity analyses (best-worst and worst-best) will be conducted under the assumption of missing not at random [43, 44]. The PHQ-9 mean scores at 3- and 12-months, the changes in SSSU scores and the proportions of mental health service use in the past year between the groups will be compared using t test and Chi square test, respectively (secondary objective #1 & #2). We will investigate if self-efficacy is a moderator in the relationships between the intervention and outcomes by including a two-way interaction term between self-efficacy and intervention status in the models (secondary objective #3). If no evidence supports moderation by self-efficacy, we will examine if self-efficacy plays a role of mediator using structural equation modeling.

As part of sex-/gender-based analysis, when analyzing the data by biological sex, we will first describe gender identity and the gender role related variables (employment status, education, income levels, and caregiving burden) to identify any gender differences and intersectionality (overlapping and interdependence among the gender role related factors) within and between the groups. In sex specific analysis, we will control the effects of gender and gender role related variables in the models. Additionally, we will evaluate the impact of the intervention on the risk of MDE by gender through stratified analysis and logistic regression modeling. As exploratory analyses, we will compare the outcomes between groups in completers, by history of MDE, and by gender identity.

### Sample size calculation

In our 2020/21 pilot study, recruitment and intervention was conducted in male and female participants, separately. Participants were assessed at baseline and 3 months post intervention. Comparing the proportions of moderate/severe MDE (having a PHQ-9 score of 15+) (31) between baseline and 3-months, the proportion of MDE in the male intervention group reduced from 15.7 to 6.2% (-9.5%), whereas the proportion in the male control group increased from 10.8 to 12.7% (+ 1.9%). The proportion in the female intervention group changed from 12.8 to 14.5% (+ 1.7%), whereas the proportion in the female control group increased from 7.4 to 17.1% (+ 9.7%). However, the increase in depressive symptoms in female intervention group (1.9%) was not statistically significant. The slight increase may be due to the fact that the pilot was conducted during the height of COVID-19 when females were more emotionally affected [47, 48]. It does not mean that the intervention is harmful as compared to the increase in the control group; it may be an indication that the intervention helped maintain the mental health of females during the pandemic, whereas the mental health of those in the control group deteriorated significantly. In the post pandemic era, we expect that females who are in the intervention group will have a lower risk of MDE than those in the control group.

Assuming the 12-month incidence of MDE in female intervention and control groups is 1.7% and 9.7%, respectively, α = 0.05 and β = 0.80, G*Power calculation [49] indicates that at least 262 female participants are needed at baseline (131 in each group). Assuming the response rate over 12 months is 80% as we achieved in our previous trial [33], 330 female participants should be recruited. We also plan to examine the moderation effect by self-efficacy. Therefore, we proposed to recruit 500 male and 500 female participants at baseline which will results in the power of 82% for the moderation analysis based on 50 Monte Carlo simulations.

## Discussion

*Current status.* At the time of submission of this manuscript, we have identified a telephone interview firm who will carry out the recruitment, screening and random selection. The next step will be to program the interview questionnaire into the CATI system. The recruitment is expected to start on November 1st, 2024.

*Potential risk and mitigation strategies.* The recruitment, data collection, and coach guidance will be undertaken via telephone or video. The research staff will not meet the participants in person. Our previous trial showed that providing individualized depression risk does not lead to psychological harms [33]. We will collect data about participants’ mental health. Some participants may feel uncomfortable answering these questions, causing psychological distress. However, we will explicitly inform the participants that participation is voluntary, and they can refuse to answer any questions or withdraw from the study at any time. The data will only be presented, shared, and published in aggregate form. Individuals with an MDE as determined by a diagnostic instrument at the time of screening are not eligible for this study; they will be recommended to seek treatment from health professionals. Through our previous trials [35, 50], we have a protocol in place for helping those who are screened as experiencing ongoing MDE or suicidal behaviours. Our pilot study showed that over 95% of participants in the intervention group agreed to receive the PDRC and the coaching session. Therefore, we do not expect issues in compliance.

We acknowledge concerns about the changes in response rates in telephone surveys due to cell phone use and telemarketing. Including eligible participants across the country will enhance the generalizability of the study. Given the vast geographic area of Canada, RDD is the only feasible method. The goal of this study is to recruit participants for a RCT, rather than selecting a representative sample. In an RCT, selection bias is not a serious concern as long as the bias is the same across the intervention and control groups [24]. To mitigate the risk, the telephone survey firm will also access the validated cellphone database. However, the use of cellphone numbers is associated with increased costs. Another potential risk of the proposed study is attrition which may incur selection bias. In previous RCT trial, the response rate at 12-month was 80% in females and 77% in males [33]. As we plan to use the same recruitment and follow-up methods, we anticipate the same response rate in the proposed RCT. Our strategies for reducing attrition will include appropriately designed introductory scripts, a minimum of nine call back attempts spaced over weekdays and times of day and provision of a $25 incentive for each completed interview.

*Knowledge translation (KT).* KT will be in the form of peer reviewed publications, conference presentations and research website dissemination. Only aggregated data will be presented. If the results of this study support the hypothesis, our goal is to implement the coach-guided PDRC in the community as a selective prevention program at Tier 1 and 2 levels through knowledge dissemination and collaboration with key knowledge users, including Public Health Agency of Canada, Mental Health Commission of Canada, Canadian Mental Health Association and mental health units of health regions. We will publish the results of the study in peer-reviewed journals, present at academic and non-academic conferences and symposia, and disseminate the key findings through social media. The authorship of the publications generated from this trial will be determined according to the BMC Medical Research Methodology authorship guidelines. No scientific writers will be used.

*In summary*, depression is a prevalent and disabling mental illness. Effective self-help can be a way to reduce the risk of depression, which is strongly supported and promoted by the WHO. The proposed study is the first RCT on using coach-guided PDRC to promote effective self-help, if successful, will lead to a novel selective prevention program that is closely aligned with the tiered mental health services model. Our prior research and pilot RCT have shown that the proposed study is highly feasible both methodologically and operationally and has great potential to generate positive results.

## Electronic supplementary material

Below is the link to the electronic supplementary material.

Supplementary Material 1

Supplementary Material 2

Supplementary Material 3

## Data Availability

No datasets were generated or analysed during the current study.

## Abbreviations

CATI: Computer assisted telephone interview
GAD: Generalized Anxiety Disorder
ITT: Intention-to-treat
KT: Knowledge translation
MDE: Major depressive episode
MHCC: Mental Health Commission of Canada
MVRP: Multivariate risk prediction
PDRC: Personalized depression risk communication
PHAC: Public Health Agency of Canada
PHQ: Patient Health Questionnaire
PI: Principal investigator
PLE: People with lived experience
RCT: Randomized controlled trial
SPOR: Strategy for patient-oriented research
TSC: Trial steering committee
WHO: World Health Organization
