# Supplementary figures and images for: The impact of a coach-guided personalized depression risk communication program on the risk of major depressive episode: study protocol for a randomized controlled trial
Source: BMC Psychiatry. 2024 Dec 18;24:916. doi: 10.1186/s12888-024-06393-9 (PMC11654057; doi:10.1186/s12888-024-06393-9)

Supplement figure #1:

Figure 1. Tiered model for mental health and addiction services


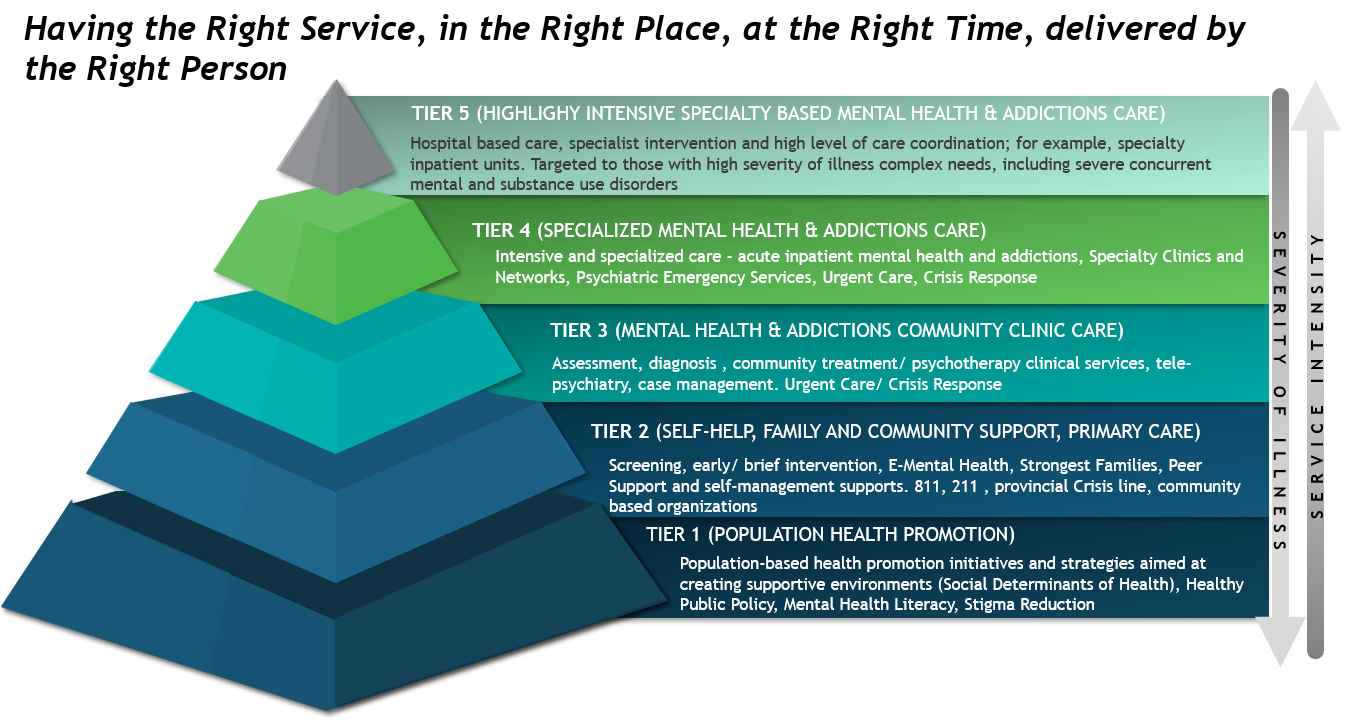

Supplement: Supplementary file 1 — Supplementary Material 1 [file 12888_2024_6393_MOESM1_ESM.docx]
