# Supplementary material for: The impact of a coach-guided personalized depression risk communication program on the risk of major depressive episode: study protocol for a randomized controlled trial
Source: BMC Psychiatry. 2024 Dec 18;24:916. doi: 10.1186/s12888-024-06393-9 (PMC11654057; doi:10.1186/s12888-024-06393-9)
Supplement: Supplementary file 3 — Supplementary Material 3 [file 12888_2024_6393_MOESM3_ESM.docx]

**Supplement file #2: Participant timeline**

|  | Study period | | | | | |
| --- | --- | --- | --- | --- | --- | --- |
|  | Enrollment | Allocation | Post-allocation | | | |
| **Time points** | -T_0_ | T_0_ (baseline) | T_1_  7 days | T_2_  3 months | T_3_  T_2_+7 days | T_4_  12 months |
| **Enrollment** |  |  |  |  |  |  |
| Eligibility screen | X |  |  |  |  |  |
| Informed consent | X |  |  |  |  |  |
| allocation |  | X |  |  |  |  |
| **Interventions** |  |  |  |  |  |  |
| Coach-guided risk communication |  |  | X | X | X | X |
| Comparison |  |  | X | X | X | X |
| **Assessments** |  |  |  |  |  |  |
| Baseline variables |  | X |  | X |  | X |
| Outcome variables |  | X |  | X |  | X |
